# Meningioma of posterolateral tentorial incisura: a case demonstration of paramedian supracerebellar transtentorial approach

**DOI:** 10.3171/2021.4.FOCVID2138

**Published:** 2021-07-01

**Authors:** Abdullah Keles, Burak Ozaydin, Mustafa K. Baskaya

**Affiliations:** Department of Neurological Surgery, University of Wisconsin School of Medicine and Public Health, Madison, Wisconsin

**Keywords:** meningioma, paramedian supracerebellar transtentorial approach, pineal region, quadrigeminal plate, tentorial incisura

## Abstract

The paramedian supracerebellar transtentorial approach allows unobstructed exposure to the quadrigeminal cistern, tectal plate, pineal region, tentorial incisura, medial basal temporal lobe, and posterior ambient cistern. The authors present a meningioma of the posterolateral tentorial incisura case in a 62-year-old male who presented with a long history of upper-extremity tremors and walking difficulties. MRI revealed supra- and infratentorial tumor extension and hydrocephalus. This approach enabled us to achieve gross-total resection without causing neurovascular injury or any postoperative neurological deficits. For each pathology, the pros and cons of various approaches should be considered based on the anatomy, vasculature, and any surrounding structures.

The video can be found here: https://stream.cadmore.media/r10.3171/2021.4.FOCVID2138.

## Transcript

### 0:23 Case Presentation

We present a case of a 62-year-old male with a long history of upper-extremity tremors and walking difficulties. Due to progressive gait problems and frequent falls, he eventually became wheelchair-bound.

### 0:34 Preoperative Imaging

MRI showed a 3 × 2.5–cm tentorial meningioma with supra- and infratentorial extensions. This caused a significant mass effect in the tentorial incisura and midbrain compression. T2-weighted MRI suggested vasogenic edema at the midbrain-tumor interface and hydrocephalus.

### 0:53 Management

After describing these findings and discussing viable treatment options with the patient and his family, we received their consent to proceed with a microsurgical excision of this meningioma using a paramedian supracerebellar transtentorial approach.

This approach allows us an unobstructed exposure to the quadrigeminal cistern, tectal plate, pineal region, tentorial incisura, as well as medial basal temporal lobe and posterior ambient cistern, through the transtentorial route.

Prior to surgery, due to hydrocephalus, we placed a right frontal ventriculostomy.

### 1:27 Patient Positioning and Skin Incision

The patient was positioned prone in a Mayfield head holder with his neck slightly flexed. The incision and trajectory of the transverse sinus were then marked with the aid of intraoperative neuronavigation.

We planned a hockey-stick incision, with the vertical segment along the midline and the horizontal segment eccentric to the left side, positioned at one-quarter above and three-quarters below the transverse sinus level. In this particular case, we chose to use a hockey-stick incision to enable a better subperiosteal dissection of the paravertebral muscles, in order to avoid postoperative muscle atrophy and pain.

The skin was prepped and draped in the usual fashion. After the skin incision, a pericranial flap was harvested for later use in dural closure. The musculocutaneous flap was elevated to expose the subperiostal bone and the craniocervical junction. Burr holes were placed on either side of the midline and just superior to the transverse sinus bilaterally. A suboccipital craniotomy was created eccentric to the left, but slightly crossing the midline in order to facilitate the widest possible left paramedian supracerebellar transtentorial approach.

### 2:38 Dural Opening

The dura was opened parallel to the transverse sinus and retracted significantly to widen the supracerebellar exposure. Crossing the transverse sinus with this craniotomy facilitates this part of the exposure. The operative microscope was then brought into the field.

Here, the viewer can see the initial microscopic exposure after the reflection and retraction of the dura. At this stage of the surgery, CSF aspiration from a small dural opening near the foramen magnum can be used to aid brain relaxation. However, since we had preoperatively placed a ventriculostomy, we did not need to release CSF in this case.

### 3:09 Arachnoid Dissection

As we deepened our dissection, we reached the quadrigeminal cistern. We then sharply opened the arachnoid covering the deep venous structures.

As we extended the arachnoid dissection inferiorly, the infratentorial portion of the yellow-tan-colored soft tumor became visible. Upon exposure of the tumor, we initiated arachnoid dissection around the tumor and the surrounding vasculature, as much as possible.

### 3:36 Tumor Sampling

After taking an initial specimen, we decided to proceed with early internal debulking since there was insufficient space to attack the tumor’s tentorial base.

After adequate internal decompression, we then coagulated the dural base of the tumor. The tentorium, in such tentorial meningioma cases, is often quite vascular and requires significant bipolar electrocautery.

### 3:58 Tentorial Incision

We then initiated a tentorial incision, which we then extended both superiorly and inferiorly until reaching the tentorial edge. Removing the portion of the tentorium that constituted the tumor base provided devascularization of the tumor base and enabled better visualization of the tumor bulk over the supratentorial side and then the tentorial incisura.

While resecting this portion of the tentorium, care must be taken to avoid injury to the posterior medial temporal lobe, branches of the posterior cerebral artery, and the temporal basal veins.

The tentorial resection that we performed along the base of the tumor had widened the surgical corridor. This enabled improved visualization and exposure of the arachnoid over the posterior medial temporal lobe–tumor interface, which then greatly facilitated our dissection. We then deepened the corridor between the subtemporal space and the tumor.

### 4:52 Tumor Resection

Continued debulking and piecemeal removal of the tumor further opened the operative corridor. We then employed sharp dissection of the arachnoid planes to further define the tumor-parenchymal interface superiorly, inferiorly, and laterally. Once the tumor had been sufficiently debulked and the cerebellar surface had been defined, and we then turned back toward the medial side and followed the arachnoid plane between the tumor and the parenchyma, eventually reaching the quadrigeminal plate.

As seen here, adequate internal debulking and piecemeal removal enabled easier and atraumatic dissection along the tumor-parenchymal interface, without causing vascular or parenchymal injury.

We then continued tumor resection by alternating internal debulking, piecemeal removal, and circumferential arachnoid dissection.

We believe that internal debulking and piecemeal removal of the tumor extent facilitates the circumferential arachnoid dissection and prevents pial or parenchymal injury over the tumor-brain interface.

As we advanced our tumor resection and circumferential dissection, we encountered the trochlear nerve, which we then dissected out of the tumor surface.

Another important point to emphasize is that neurosurgeons must be aware of a tumor’s pial feeders while performing circumferential dissections. Pial feeders may emerge as side feeders from en passant arteries. During coagulation and dividing of these side feeders, special care must be taken not to injure any en passant arteries that perfuse the normal brain parenchyma and ensure that only the tumor feeders are taken.

As seen in this segment, a pial feeder emerging from a superior cerebellar artery branch as a side feeder to the tumor is being coagulated and divided right at the point it enters the tumor.

The tumor was now essentially avascular. Gentle dissection of the arachnoid plane at the interface between tumor and normal parenchyma continued to define our operative plane. Gentle dissection along the arachnoid plane helped avoid pial and parenchymal injury, especially injury to the quadrigeminal plate and the colliculi. With soft tumors we like to use low-intensity ultrasonic aspiration to aid internal debulking and avoid injury to the surrounding neurovascular structures and the parenchyma. After adequate internal debulking of the tumor, we then completed the circumferential dissection, since we were then able to remove the remainder of the tumor en bloc.

Here we can see the quadrigeminal plate after completing the tumor removal. Through the operative corridor, we now have an unobstructed view of the medial temporal lobe. Hemostasis was then achieved.

### 7:26 Dural, Craniotomy, and Skin Closure

The dura was then closed using the previously harvested pericranial graft in a watertight fashion. The cranial bone was reaffixed, and multilayered closure of the subcutaneous layers and the skin was performed.

### 7:37 Postoperative Imaging

Postoperative MRI confirmed gross-total resection of the tumor without any parenchymal injury. Histopathology of the tumor was confirmed as WHO grade I transitional meningioma.

### 7:47 Postoperative Course

The patient woke up without any new deficits, and his postoperative course was uneventful. He was discharged to a rehabilitation center. Preoperative symptoms and the hydrocephalus were resolved postoperatively. At his 5-year follow-up, the patient was neurologically intact and functioning independently without any residual or recurrent meningioma.

### 8:07 Conclusion

In conclusion, intracranial pathologies in the tentorial incisura, quadrigeminal cistern, and pineal regions can be approached via posterior interhemispheric, suboccipital transtentorial, median supracerebellar infratentorial, paramedian supratentorial, and subtemporal approaches.^1–3^

During tumor resection in this deep region, profuse bleeding may be encountered which can be challenging to control. Such bleeding can decrease the surgeon’s view and, therefore, can lead to inadvertent injury of critical neurovascular structures. Thus, it is essential to achieve early interruption of the blood supply to the tumor, in order to enable safe surgery.

In the present case, we elected to utilize the paramedian supracerebellar transtentorial approach because this approach provides early visualization of the tentorial base, which facilitates prompt devascularization. This approach also avoids traction injuries to the vein of Labbé and the veins coursing along the temporal base, when compared to subtemporal approaches. This approach also protects the medial basal temporal lobe and occipital lobe, especially the primary visual cortex located in the calcarine fissure, which may be injured with subtemporal and occipital interhemispheric approaches, respectively.^4,5^ In addition, the paramedian supracerebellar transtentorial approach provides a direct operative corridor toward the tumor, without any interference with deep venous vasculature, as may be the case with the supratentorial approaches. Moreover, this approach also enables complete resection of the involved tentorium, which is critical in preventing recurrence.^1^

Finally, in comparison to the median supracerebellar approach, the paramedian supracerebellar approach provides a better angle while reaching the quadrigeminal cistern and pineal region and avoids encountering midline bridging veins.^6–8^ However, with the paramedian approach, exposure of the contralateral structures might be somewhat limited.

In conclusion, for each pathology, both the pros and cons of various approaches should be considered based on the anatomical location, vasculature, and any surrounding critical structures.

## Author Contributions

Primary surgeon: Baskaya. Editing and drafting the video and abstract: all authors. Critically revising the work: all authors. Reviewed submitted version of the work: Baskaya, Keles. Approved the final version of the work on behalf of all authors: Baskaya. Supervision: Baskaya.
